# New Insights about Antibiotic Production by *Pseudomonas aeruginosa*: A Gene Expression Analysis

**DOI:** 10.3389/fchem.2017.00066

**Published:** 2017-09-15

**Authors:** Bárbara Gionco, Eliandro R. Tavares, Admilton G. de Oliveira, Sueli F. Yamada-Ogatta, Anderson O. do Carmo, Ulisses de Pádua Pereira, Roberta T. Chideroli, Ane S. Simionato, Miguel O. P. Navarro, Andreas L. Chryssafidis, Galdino Andrade

**Affiliations:** ^1^Microbial Ecology Laboratory, Department of Microbiology, Universidade Estadual de Londrina Londrina, Brazil; ^2^Molecular Biology Laboratory, Department of Microbiology, Universidade Estadual de Londrina Londrina, Brazil; ^3^Department of General Biology, Institute of Biologic Sciences, Universidade Federal de Minas Gerais Belo Horizonte, Brazil; ^4^Laboratory of Fish Bacteriology, Department of Preventive Veterinary Medicine, Universidade Estadual de Londrina Londrina, Brazil; ^5^Laboratory of Veterinary Toxicology, Department of Preventive Veterinary Medicine, Universidade Estadual de Londrina Londrina, Brazil

**Keywords:** bioactive compounds, RNA-seq, antibiotic, Organometallic Antibiotic

## Abstract

The bacterial resistance for antibiotics is one of the most important problems in public health and only a small number of new products are in development. Antagonistic microorganisms from soil are a promising source of new candidate molecules. Products of secondary metabolism confer adaptive advantages for their producer, in the competition for nutrients in the microbial community. The biosynthesis process of compounds with antibiotic activity is the key to optimize their production and the transcriptomic study of microorganisms is of great benefit for the discovery of these metabolic pathways. *Pseudomonas aeruginosa* LV strain growing in the presence of copper chloride produces a bioactive organometallic compound, which has a potent antimicrobial activity against various microorganisms. The objective of this study was to verify overexpressed genes and evaluate their relation to the organometallic biosynthesis in this microorganism. *P. aeruginosa* LV strain was cultured in presence and absence of copper chloride. Two methods were used for transcriptomic analysis, genome reference-guided assembly and de novo assembly. The genome referenced analysis identified nine upregulated genes when bacteria were exposed to copper chloride, while the De Novo Assembly identified 12 upregulated genes. Nineteen genes can be related to an increased microbial metabolism for the extrusion process of exceeding intracellular copper. Two important genes are related to the biosynthesis of phenazine and tetrapyrroles compounds, which can be involved in the bioremediation of intracellular copper and we suggesting that may involve in the biosynthesis of the organometallic compound. Additional studies are being carried out to further prove the function of the described genes and relate them to the biosynthetic pathway of the organometallic compound.

## Introduction

The increasing incidence of bacterial resistance against antibiotic compounds is a global problem in patients within a hospital environment. The multi-drug resistant (MDR) microorganisms have a significant impact in the mortality and in the hospitalization time of patients, increasing the cost of treatments associated with bacterial infection control (Rundramurthy et al., 2016). Currently, the high level of MDR bacteria dissemination is a worldwide major public health problem and the challenge is to find new antibiotics to control infections caused by these microorganisms (Bassetti et al., 2013). In the 90's, the pharmaceutical industry did not invest in new antibiotic products for many reasons, such as the time and cost for the development of new antibiotics. Due to a number of factors, antimicrobial resistance may emerge shortly after drug utilization and the future is uncertain, as some agencies estimate that none of the antibiotics currently in use will be effective against MDR bacteria in less than 10 years (Carlet et al., 2012).

The microbial community of soil was the source of many antimicrobials (Newman and Cragg, 2007). It is largely known that competing microorganisms produce some compounds to inhibit other microbial growth, using this mechanism as a survival strategy (Andrade, 2004). Therefore, to understand the interactions between microbial populations in the microcosm where they live and compete will certainly help to identify antagonism, and the bioactive compounds involved in these relations may be employed against bacterial infections (Fernandes, 2015).

Bacteria can produce thousands of compounds with bacteriostatic or bactericide activity, with potential use in the control of MDR bacteria (Butler et al., 2013). These compounds are usually products of secondary metabolism and would be involved in bacteria survival (Bérdy, 2005). Multiple studies described substances with bactericide or antifungal activity produced in the secondary metabolism of microorganisms, which could be applied in the management of human, animal and plant diseases (Stierle and Stierle, 2015; Depoorter et al., 2016; Thi et al., 2016).

Previous studies at the Microbial Ecology Laboratory showed that *Pseudomonas aeruginosa* LV strain produced an organometallic compound with antimicrobial activity against MDR bacteria, including *Klebsiella pneumoniae* carbapenemase producer KPC (*K. pneumoniae* carbapenemase) (Kerbauy et al., 2016), and *Staphylococcus aureus* methicillin resistant (Cardozo et al., 2013). Additionally, the same strain produced a Phenazine-1-Carboxylic Acid (even if in low quantity) which shown antifungal potential against *Botrytis cinerea* (Simionato et al., 2017). Apparently, the copper chloride induces the production of this compounds, since no antimicrobial activity can be detected in the supernatant of *P. aeruginosa* strain LV cultured in its absence (De Oliveira et al., 2016).

In this context, the objective of this work was to find the expressed genes related to the production of the bioactive organometallic compound in the presence of copper and related to the biosynthesis pathway in the *P. aeruginosa* strain LV using the transcriptomic approach.

## Materials and methods

### Culture of bacterial strain

*P. aeruginosa* LV strain was cultured in nutrient broth at 28°C for 24 h in the presence or absence of copper chloride (0.1 g L^−1^), according to patent (PI0803350-1 – INPI 12/09/20092008; http://www.inpi.gov.br). The cells were recovered by centrifugation in a Jouan CR3 equipment (9,000 rpm/20 min, 4°C), resuspended in TRIZOL (INVITROGEN) and kept at −80°C. The confirmation of the antibiotic activity of the supernatants was confirmed as described elsewhere (De Oliveira et al., 2016). Both cultures were performed in biological triplicate (three samples for each condition, corresponding to three experiments separated spatially and temporally).

### Total RNA extraction and ribosomal RNA depletion

The total RNA was isolated using the illustra RNAspin kit™ (Ge Healthcare, UK) and ribosomal RNA depletion was performed using the RiboMinus™ transcriptome Kits (TermoFisher Scientific), followed the manufacturer's recommendation. The integrity of RNA was checked in agarose gel (1.5%) in a denaturant condition and the amount of RNA was determined using a spectrophotometer (Figures S7, S8) (BioTek Sinergy HT).

### cDNA library construction and sequencing

The construction of library was carried at Biotechnology and Molecular Markers Laboratory in the Universidade Federal de Minas Gerais (Belo Horizonte, MG, Brazil). The rRNA-depleted RNA (1 μg) was used to build cDNA library using the kit TruSeq® RNA Sample Preparation v.2 (Illumina, USA). The libraries were quantified with KAPA Library Quantification Illumina® (KAPA Biosystems) by quantitative PCR. The libraries were normalized to the concentration of 4 nM and sequencing was performed with MiSeq sequencer (Illumina) using MiSeq Reagent v3 (600 cycles) paired-end 2 × 300.

### Detection of differentially transcribed genes

#### Reference-guided genome analysis

Due the absence of complete genome of *P. aeruginosa* LV strain, a more close genome for used for this analysis. For that, ten complete genomes of *P. aeruginosa* was randomly choiced for read mapping step. For the reason of more percentage of read mapped, the reference genome of *P. aeruginosa* deposited at the NCBI accession number NC_002516.2 was picked for Reference-guided genome analysis. The quality of the reads was analyzed in the platform of CLC Genomics Workbench version 8.5.1 (CLC bio, Denmark). The analysis was carried out using FastQC, which showed a high quality of reads and the use of filter was discarded, in order to guarantee the whole information about the sequences. The data was analyzed by CLC Genomics Workbench platform to quantify the expression by RPKM values (Reads per Kilobase per Million of Mapped Reads). Empirical analysis of DGE (Robinson and Smyth, 2008) hosted in the CLC platform was used to determine the differentially transcribed genes between copper-growth conditions. The genes were normalized and used for the dispersion analysis. The differential transcripts in presence of copper were identified and filtered applying the corrector Fold Change > than 1.5 and *p* < 0.001.

#### *De novo* assembly of transcripts

The analysis of raw RNA-seq data was performed using the Rockhopper 2 software platform (Tjaden, 2015). The FASTQ files were uploaded directly to the platform using a paired-end library approach. De novo assembly of bacterial transcriptomes was done using the software default settings. Rockhopper 2 normalized each RNA-seq data set using upper quartile normalization and employed a modified RPKM value and foldchange to quantify transcript abundance levels. The difference in expression was evaluated based on the results of transcript foldchange. The negative binomial distribution was used as the statistical model in order to detect significance levels that indicated the probability of observing difference in a transcript expression level under presence and absence of copper chloride. Resulting *p*-values were corrected and *q*-values were employed to control false positives, using the Benjamini-Hochberg procedure (Tjaden, 2015). In summary, Rockhopper 2 estimated the variance of a transcript expression, using local regression to obtain a smooth estimate of the variance, and performed a statistical test to determine whether a transcript presented different level of expression under the presence or absence of copper.

### Identification of functional genes differentially expressed

The transcripts differentially expressed in presence of copper were analyzed using BLAST (Basic Local Alignment Search Tool), INTERPRO (Protein Sequence Analysis and Classification), UNIPROT (Universal Protein Resource) e PFAM (Protein Domain) databases, separately and manually classified according to the similarity of sequences description and function.

## Results

The gene expression analysis of *P. aeruginosa* LV strain was realized in two different growth conditions, in the presence or absence of copper, using two bioinformatics strategies, genome reference-based and de novo assembly, with the identification of differently expressed genes and their putative function. The results suggest that some genes are involved in the biosynthesis of the organometallic compound with antimicrobial activity and other genes are related with the detoxification, or influx reduction, of copper inside the bacterial cells.

### Reads obtention

**Figure 1:**
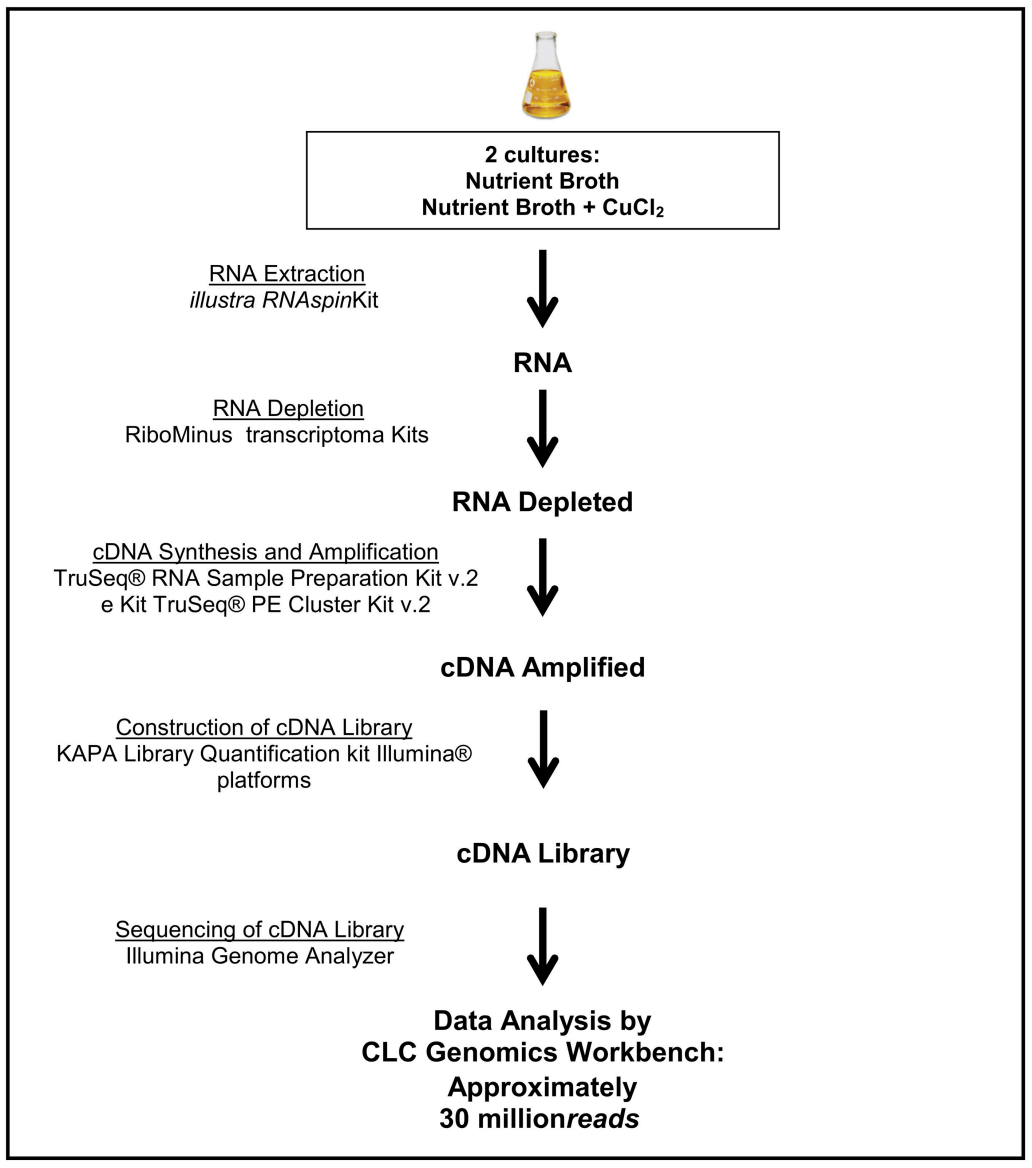
Workflow of RNA extraction steps, RNA depletion, cDNA synthesis, construction, and sequencing of genomic libraries.

The generated RNA-Seq library was composed by 30 million reads, obtained by RNA extraction and depletion and synthesis and sequencing of resulting cDNA (Figure 1). The reads were separated in two groups, corresponding to the *P. aeruginosa* strain LV culture conditions (presence or absence of copper chloride). The first group (absence of copper) was composed of 14,783,618 reads, and the second group (presence of copper) contained 15,091,252 read (Table 1). The triplicates of each group were analyzed together and separately, generating similar results.

**Table 1:** The table shows the number and percentage of mapped and unmapped *reads* the triplicates of each treatment.

		**Paired-mapped reads**		**Unmapped reads**		**Total^a^**	
		**Reads**	**%**	**Reads**	**%**	**Reads**	**%**
Treatment A (no cooper)	A1	4,080,686	77.29	1,060,775	20.09	5,279,934	100
	A2	3,077,138	62.52	1,723,699	35.02	4,921,736	100
	A3	1,753,948	38.28	2,694,837	58.81	4,581,948	100
Treatment B (presence of cooper)	B1	3,081,482	63.97	1,592,615	33.06	4,816,716	100
	B2	3,511,134	68.74	1,469,480	28.77	5,107,976	100
	B3	2,878,158	55.71	2,164,010	41.88	5,166,560	100

a *The sum of mapped and unmapped reads values does not correspond to the total shown because the reads mapped reads in broken pairs were hidden from the table*.

### Measure of expression levels and differential expression analysis in guided genome

**Figure 2:**
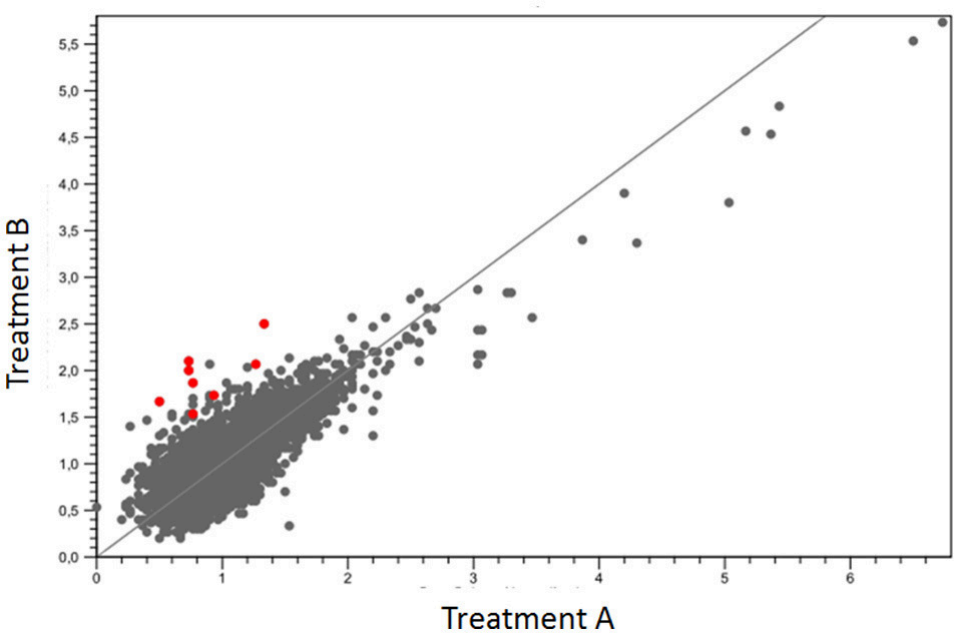
Dispersion of the expressed genes between the treatments when compared to the reference genome PA01. Red dots indicate hyperexpressed genes with lower levels of significance.

The mean covering of each treatment was greater than 100× on the reference genome. Approximately 63% of reads from group one and 65% of group two were mapped (Table 1). The quantification of gene expression used the values of RPKM. The mapping data obtained between the different growth conditions were compared for determining the levels and differences in the gene expression. Nine genes were significantly upregulated (Figure 2), suggesting their relation with the biosynthesis of the bioactive organometallic compound, which only occurs in the presence of copper. On the other hand, four genes related to copper efflux were downregulated in cells cultured in the presence of copper.

### Functional analysis

Upregulated genes were filtered and classified in functional categories, based on the similarity of sequence description for reference-guided genome analysis (Table 2) and de novo assembly (Table 3). Downregulated genes were also identified (Table 4).

**Table 2:** Functional categories of overexpressed genes with statistical significance for the four analyzed databases and their access in Genome Guided Analysis.

**GENOME GUIDED ANALYSIS**								
**Genes**	**BLAST**		**INTERPRO**		**UNIPROT**		**PFAN**	
	**Access**	**Description**	**Access**	**Description**	**Access**	**Description**	**Access**	**Description**
PA2691	NP_251381.1	Hypothetical protein	NP_251381.1	None predicted	Q9I0F1	Uncharacterized protein	119AC74A-4308-11E6-911D-1D4195374F7B	Pyridine nucleotide-disulphide oxidoreductase
PA3521	NP_252211.1	Hypothetical protein	NP_252211.1	Outer membrane efflux protein	Q9HY88	Probable outer membrane protein	8C838970-4307-11E6-8525-AA236FECAC22	Outer membrane efflux protein
PA3523	NP_252213.1	Resistance-nodulation-cell division (RND)/efflux membrane fusion protein	NP_252213.1	RND efflux pump, membrane fusion protein	A0A0H3R045	Efflux transporter, RND family, MFP subunit	E7D46600-4307-11E6-AB34-2E4195374F7B	Barrel-sandwich domain of CusB or HlyD membrane-fusion
PA3574a	YP_008719768.1	Copper chaperone CopZ	YP_008719768.1	None predicted	A0A0H3QZK8	Uncharacterized protein	96066EEE-4308-11E6-80EB-32246FECAC22	HMA domain (heavy-metal-associated domain)
PA3920	NP_252609.1	Metal transporting P-type ATPase	NP_252609.1	P-type ATPase	A0A0H3R5R9	Heavy metal translocating P-type ATPase	BF3D3A22-4308-11E6-8366-95635F09777C	HMA domain (heavy-metal-associated domain)
PA4141	NP_252830.1	Hypothetical protein	NP_252830.1	None predicted	A0A0E1AMZ0	Uncharacterized protein	E93E4DAC-4308-11E6-A9B7-1C4195374F7B	We did not find any Pfam-A matches to your search sequence
PA4782	NP_253470.1	Hypothetical protein	NP_253470.1	None predicted	A0A0E1AZX2	Uncharacterized protein	0F91632C-4309-11E6-A6CF-1D4195374F7B	We did not find any Pfam-A matches to your search sequence
PA4878	NP_253565.1	Transcriptional regulator	NP_253565.1	None predicted	A0A0E1B2A9	Transcriptional regulator	29269938-4309-11E6-B71C-1C4195374F7B	MerR HTH family regulatory protein
phzA2	NP_250590.1	Phenazine biosynthesis protein PhzA	NP_250590.1	Phenazine biosynthesis protein A/B	V6AFZ3	Phenazine biosynthesis protein phzA 2	3E312AE6-4309-11E6-9A91-32246FECAC22	Phenazine biosynthesis protein A/B

**Table 3:** Functional categories of overexpressed genes with statistical significance for the four analyzed databases and their access, in *De novo* analysis.

***DE NOVO*** **ANALYSIS**								
**Genes**	**BLAST**		**INTERPRO**		**UNIPROT**		**PFAN**	
	**Access**	**Description**	**Access**	**Description**	**Access**	**Description**	**Access**	**Description**
BAV0296	WP_012415998.1	Glutamyl-tRNA reductase	IPR000343	Tetrapyrrole biosynthesis, glutamyl-tRNA reductase	Q2KZZ5	Catalyzes the NADPH-dependent reduction of glutamyl-tRNA(Glu) to glutamate 1-semialdehyde (GSA)	PF00745	Glutamyl-tRNAGlu reductase, N-terminal domain
	JFBC01000420.1	Hypothetical protein BC89_12120		None predicted	A0A136QJ84	Uncharacterized protein		We did not find any Pfam-A matches to your search sequence
RPII		DNA-directed RNA polymerase II subunit RPB1; RNA polymerase II subunit B1; DNA-directed RNA polymerase III largest subunit	IPR006592	RNA polymerase, N-terminal	P14248	DNA-directed RNA polymerase II subunit RPB1	PF04997	RNA polymerase Rpb1
Mak	NP_037268.1	Serine/threonine-protein kinase MAK; Male germ cell-associated kinase	IPR011009	Protein kinase-like domain; Protein kinase, ATP binding site; Serine/threonine-protein kinase, active site	P20793	Serine/threonine-protein kinase MAK	PF00069	Protein kinase domain
agaA	AF121273.1	Alpha-agarase	IPR005084	Carbohydrate binding module family 6	Q9LAP7	Alpha-agarase	PF03422	Carbohydrate-binding module
PA4747	NP_253435.1	Preprotein translocase subunit SecG	IPR004692	Preprotein translocase SecG subunit	Q9HV52	Involved in protein export. Participates in an early event of protein translocation	PF03840	Preprotein translocase SecG subunit
atg4	XP_002563384.1	Probable cysteine protease atg4; autophagy-related protein 4	IPR005078	Peptidase family C54	A7KAL5	Probable cysteine protease atg4	PF03416	Peptidase family
PA2853	NP_251543.1	Outer membrane lipoprotein OprI	IPR021793	Alanine-zipper, major outer membrane lipoprotein	P11221	Major outer membrane lipoprotein	PF11839	Alanine-zipper, major outer membrane lipoprotein
TIM21		Mitochondrial import inner membrane translocase subunit TIM21	IPR013261	Mitochondrial import inner membrane translocase subunit Tim21	Q4HZ95	TIM21	PF08294	TIM21
FAM120A	NP_001028440.2	Constitutive coactivator of PPAR-gamma-like protein 1; Oxidative stress-associated Src activator	IPR026784	Constitutive coactivator of PPAR-gamma	Q6A0A9	Constitutive coactivator of PPAR-gamma-like protein 1		We did not find any Pfam-A matches to your search sequence
mug8	NP_593774.1	Meiotically up-regulated gene 8 protein	IPR012965	DUF1708	Q10326	Meiotically up-regulated gene 8 protein	PF08101	DUF1708
AFUA_3G00590		Asp-hemolysin	IPR009413	Hemolysin, aegerolysin type	Q00050	Asp-hemolysin	PF06355	Aegerolysin

**Table 4:** Functional categories of down-expressed genes, with statistical significance for the four analyzed databases and their access, in both Analysis: Genome Guided and *De novo*.

**GENOME GUIDED ANALYSIS**								
	**BLAST**		**INTERPRO**		**UNIPROT**		**PFAN**	
**Gene**	**Access**	**Description**	**Access**	**Description**	**Access**	**Description**	**Access**	**Description**
PA0202	OPE24314.1	Amidase	OPE24314.1	Amidase	A0A1F0J5G9	Amidase	PDOC00494	Amidase
PA0203	AAT51424	MULTISPECIES: ABC transporter *[Pseudomonas]*	WP_034003826.1	None predicted	A0A069QCF8	ABC transporter	PF13416	Extracellular solute binding proteins.
PA0204	WP_034003826	ABC transporter permease	IPR000515	ABC transporter type 1, transmembrane domain MetI-like	A0A1F0J4R9	ABC transporter permease	PF00528	Binding-protein-dependent transport system inner membrane component
PA0205	WP_003106180	ABC transporter permease	–	None predicted	Q9I6T3	Probable permease of ABC transporter	PF00528	Binding-protein-dependent transport system inner membrane component
**DE NOVO ANALYSIS**								
LYS20	NP_010099.1	Homocitrate synthase, cytosolic isozyme	IPR000891	Pyruvate carboxylase	P48570	Homocitrate synthase, cytosolic isozyme	PF00682	HMGL-like
KUJ92327	LGEP01000013.1	hypothetical protein XD38_0062	–	None predicted	A0A101DB66	Uncharacterized protein	–	We did not find any Pfam-A matches to your search sequence

## Discussion

### Reference-guided genome analysis

Three upregulated genes (PA2691, PA4141, and PA4782) were not functionally characterized in all data banks used and were classified as hypothetical proteins. Other three upregulated genes were related to the transport of metallic ions (PA3521, PA3523, and PA3920) and one gene (PA3574a) was associated with chaperones group linked with copper. The gene (PA4872) was classified as a transcript regulator and gene (phzA2) was related to the phenazine biosynthesis.

Three hyperexpressed genes were related to the efflux system (PA3521, PA3523, and PA3920) and one gene (PA3574a) was related to a copper-chaperone, the CopZ. The PA3920 gene is related to the transport of heavy metals in *Pseudomonas* sp. and belongs to the P1B-1 subgroup of the carrying family P-type ATPases. In this system, the copper efflux occurs in the presence of ATP, when copper chaperone translocate the cation and link in a carrying protein, moving the complex through the membrane, to outside the bacterial cell (González-Guerrero et al., 2010). The *P. aeruginosa* LV strain efflux system was triggered by the presence of copper chloride, with hyperexpression of genes related to this mechanism.

The PA4878 gene belongs to a MerR family, which includes regulator proteins involved in the activation of the efflux system, in response to an inductor (Liao et al., 2013). The high expression values of this gene observed in copper-stimulated *P. aeruginosa* LV strain may be related to a set of mechanisms working to eliminate the metal from within the cell.

The downregulated genes were related to the ABC transporter (PA203), ABC transporter permease (PA204 and PA205) and an amidase (PA202). The ABC transporter system is composed of an outer membrane carrier, a membrane-fusion protein and an outer-membrane porin. These three components assemble into a complex spanning both membranes and providing the conduit for the translocation of unfolded polypeptides, as well as ions and various other substances into bacteria (Morgan et al., 2017). The downregulation of the ABC transporter and permease in the *P. aeruginosa* LV strain reveals a strategy for blocking the copper influx. Similar data were found by Li et al. (2009) in copper-resistant *Pseudomonas* spp. The reduction of the amidase gene is related to the decrease of the expression of porins, since they are involved in the entrance of polysaccharides into the bacterial cell.

The phenazine family are nitrogenous heterocycle compounds with redox activity, produced by fluorescent pseudomonads. The phenazines are small molecules that can penetrate different types of cells, including prokaryotes. Many phenazines present antimicrobial activity, suppressing pathogenic microorganisms of humans, animals and plants, also playing an important role in the survival strategies of bacteria in the environment (Chen et al., 2014; Briard et al., 2015; Dasgupta et al., 2015; Garrison et al., 2016; Morrison et al., 2016). The *P. aeruginosa* LV strain is a great producer of phenazine carboxylic acid (PCA).

The biosynthesis of phenazine in *P. aeruginosa* is realized by two seven-gene operons, phz1 (phzA1B1C2D1E1F1G1) and phz2 (phzA2B2C2D2E2F2G2). According to Li et al. (2011), there is a combined regulation of these two operons in the biosynthesis of PCA. The first one (phzA2-G2 gene) produces small amount of PCA and the second one (the phzA1-G1 gene) produces a large amount of PCA. The expression of two operons working together optimizes the PCA biosynthesis, through a positive feedback mechanism. The operon phz2 produces a basal amount of PCA, self-inducing the same operon to keep the biosynthesis process and triggering the transcription of operon phz1, amplifying PCA production.

In the present study, the hyperexpression of phzA2 gene belonging of phz2 operon was observed. Among the nine-upregulated genes identified, the phzA2 is the only one related to a biosynthesis pathway. The proposition is that the augmented expression of phzA2 gene stimulates the production of PCA and, once there is PCA in the cytoplasm, it activates its own biosynthesis process.

**Figure 3:**
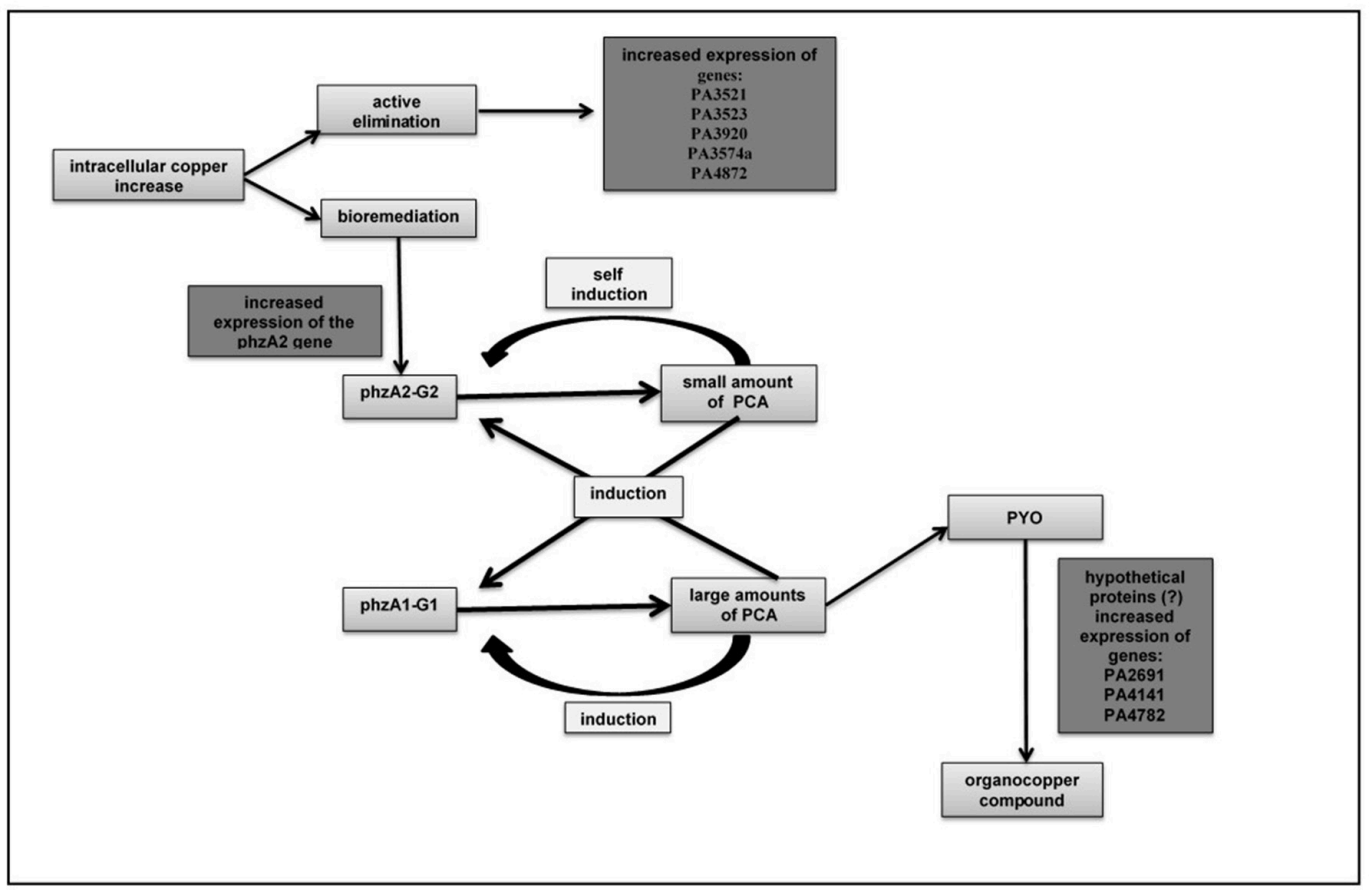
An integrated relationship of the hyperexpressed genes when there is an increase of copper in the culture medium and the possible hypotheses of its expulsion: active elimination and bioremediation.

Many studies carried out in the Microbial Ecology Laboratory demonstrated that, in absence of copper, the *P. aeruginosa* LV strain produces pyoverdine (PYO) from PCA, suggesting that the PCA is a precursor for PYO biosynthesis. However, when copper is present, the LV strain produces PCA and the PYO formed helps the bacteria to bioremediate the copper, forming the organometallic compound (Figures S1–S6) with C, Cu, S and O (Figure S9) and N (Figure S10). Possibly, the three hypothetical proteins detected are involved in the biosynthesis of PYO, using PCA as precursor (Figure 3). Further studies will be carried out to better understand such process and to confirm this hypothesis.

### *De novo* assembly

In this analysis, twelve upregulated genes and two downregulated genes were identified. In relation to upregulated genes, one gene was not functionally characterized and was classified as a hypothetical protein. Seven upregulated genes were related to increased protein synthesis (RPII, MaK, agaA, PA474, atg4, TIM21, mug8) and two genes related to oxidative stress (FAM120A and AFUA_3G00590). The PA2853 gene was related to an outer membrane lipoprotein (OprI) and the BAV0296 gene (glutamyl-tRNA reductase) was related to the synthesis of tetrapyrroles compounds. From the downregulated genes, one was related to homocitrate synthase, LYS20, and one was an uncharacterized protein, KUJ92327.

Copper is a cofactor for many important enzymes, in eukaryotes and prokaryotes and it is essential for almost all organisms. However, the excess copper can also be harmful due to its redox activity and the ability of binding to sites of other metals, particularly iron-sulfur clusters, leads to an increased microbial metabolism, related to the stress generated by the ion inside the organism (Vita et al., 2016). Seven genes related to increased protein synthesis were detected in this study. From those, five can be detected in prokaryotes: the RPII gene (a RNA-polymerase); MaK (protein kinase) related to many cellular processes, including metabolism, transcription, cell cycle progression, cytoskeletal rearrangement and cell movement, apoptosis, and differentiation; agaA (carbohydrate binding module family 6), which binds to specific ligands, such as cell-surface-attached carbohydrate substrates for galactose oxidase and sialidase; PA474 (preprotein translocase subunit SecG), involved in protein export and translocation and atg4 (peptidase family C54), a proteolytic enzyme that hydrolyses peptide bonds using the thiol group of the cysteine residue as a nucleophile.

Another two genes may be related to increased protein synthesis and cell division, TIM21 and mug8. These genes have been described in eukaryotes and are responsible for the translocation of transit peptide-containing proteins across the mitochondrial membrane and role in bud formation, respectively. This may help the bacteria to maintain the cellular division in the presence of cooper.

The gene FAM120A (oxidative stress-associated Src activator), a critical component of the oxidative stress-induced survival signaling in human, and AFUA_3G00590 (asp-hemolysin), expressed during bacterial sporulation, were also detected. Furthermore, the OprI, the major outer membrane lipoprotein of *P. aeruginosa*, presented increased expression (Basto et al., 2014). The upregulation of these genes can be explained. High levels of copper are toxic to bacteria and lead to cell damage, by generating reactive oxygen species, affecting the function of proteins or inactivating the enzymes (Li et al., 2009) and hence those overexpressed proteins act on protecting the cell against such damage.

The tetrapyrrole compounds are molecules that have four rings of the pyrrole type, generally linked together by single-atom bridges, between the alpha positions of the five-membered pyrrole rings. They function as a metal-binding cofactor in many important enzymes, proteins and pigments, such as heme, chlorophyll, cobalamine (vitamin B12), siroheme and cofator F430 (Schulze et al., 2006; Schalk and Cunrath, 2016) may suggest its participation in cooper insertion in the bioactive compound produced by LV strain. The Glutamyl-tRNA reductase is an important enzyme involved in this process. The super-expression of this enzyme detected in our study reaffirms our hypothesis that the compound with antimicrobial activity comes from the biosynthesis of pigments produced by *P. aeruginosa* strain, since the increase of tetrapyrrole can be related to the binding of copper on these pigments.

The overexpressed genes detected were different in the two methods applied in this study. Still, most of them are related to the stress in the cell caused by copper and its elimination. Furthermore, in the reference-guided genome analysis, an increase in phzA2 gene expression was found (related to phenazine biosynthesis) whilst an increased expression of the BAV0296 gene was detected in the *de novo* analysis (related to the production of tetrapyrrole compounds). Although the results are different, there is a complementarity between them, since the increase in expression of these genes, related to the pigments produced by the *P. aeruginosa* strain, can also be related to the production of the organometallic compound with antimicrobial activity, as a mechanism of bioremediation of intracellular copper. Additional studies are being carried out with these genes to confirm such hypothesis.

Regarding the down regulated gene LYS20, related to homocitrate synthase, it is known that this enzyme participates in lysine biosynthesis and pyruvate metabolism. The homocitrate synthase catalyzes the first step in lysine biosynthesis in many fungi and certain Archaea (Bulfer et al., 2009). The down expression of this gene can be related to a decreased cell anabolism, caused by cellular stress derived from excess intracellular copper.

## Conclusion

The gene expression analysis of the *P. aeruginosa* LV strain, in presence of copper chloride, suggests that the excess of copper forces the microorganism to eliminate it through different pathways. The first way of elimination is related to the hyperexpression of efflux systems, capable of expelling the metal into the periplasmic space or to the extracellular environment. A second hypothesis of ion elimination is directly related to the synthesis pathway of the bioactive compound, where the microorganism is able to bioremediate the accumulated copper inside the cell. This elimination may be associated with a deviation of the metabolic route of pigments produced by the strain, possibly bioremediating the copper and forming the compound with antimicrobial activity. The downregulation of ABC transporter genes (related to ion transport) can be a mechanism to avoid the excess of copper influx to bacteria, which would guarantee the microorganism survival. Studies are being designed and carried out to confirm the hypotheses suggested in the present study.

## Author contributions

BG, ET, SY, AdO, and GA designed the study protocol, and participated in its design and coordination. BG and ET validated and performed the experiment. AdC performed the Pseudomonas aeruginosa LV strain sequencing. UP performed and interpreted de Novo analysis. AS and MN produced and purified the compounds produced by the LV strain. BG, UP, AS, AC, and GA contributed to drafting the manuscript and/or critically revising the paper and intellectual content.

### Conflict of interest statement

The authors declare that the research was conducted in the absence of any commercial or financial relationships that could be construed as a potential conflict of interest.
