# Supplementary figures and images for: New Insights about Antibiotic Production by Pseudomonas aeruginosa: A Gene Expression Analysis
Source: Front Chem. 2017 Sep 15;5:66. doi: 10.3389/fchem.2017.00066 (PMC5605626; doi:10.3389/fchem.2017.00066)

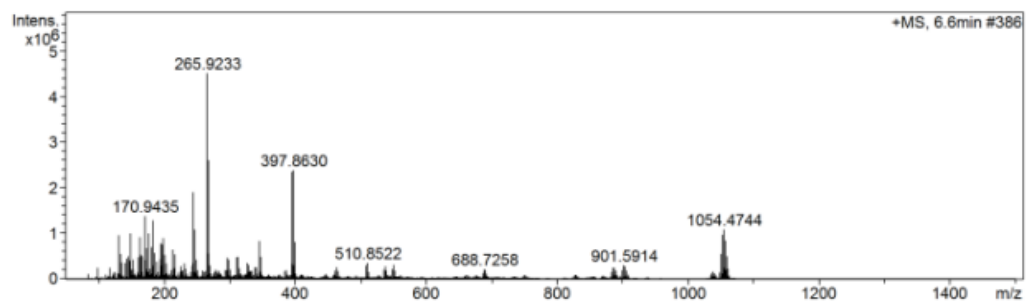

**Figure S1.** ESI-MS spectrum of compound **1** (positive mode).

Supplement: Supplementary file 1 [file Image1.PDF]

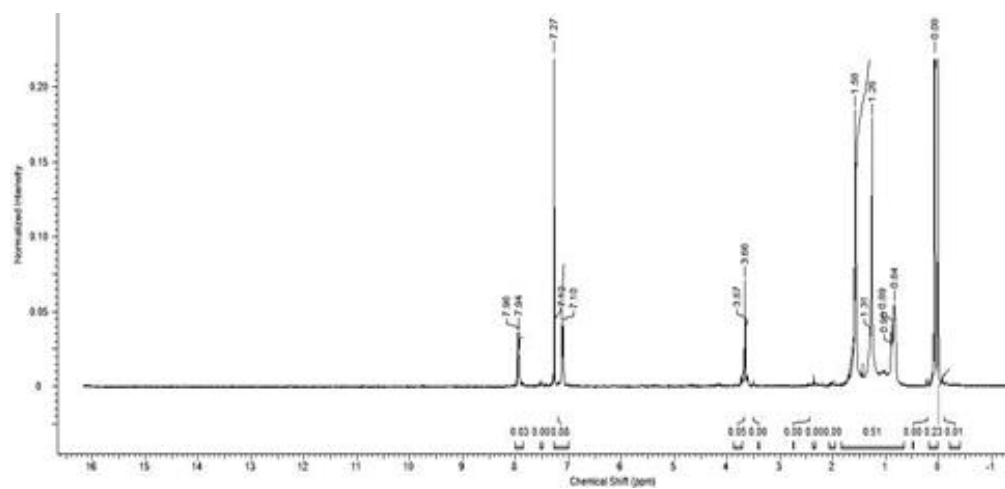

**Figure S2.**  $^1\text{H}$  NMR spectrum of compound **1** (300 MHz,  $\text{CD}_3\text{OD}$ ).

Supplement: Supplementary file 2 [file Image2.pdf]

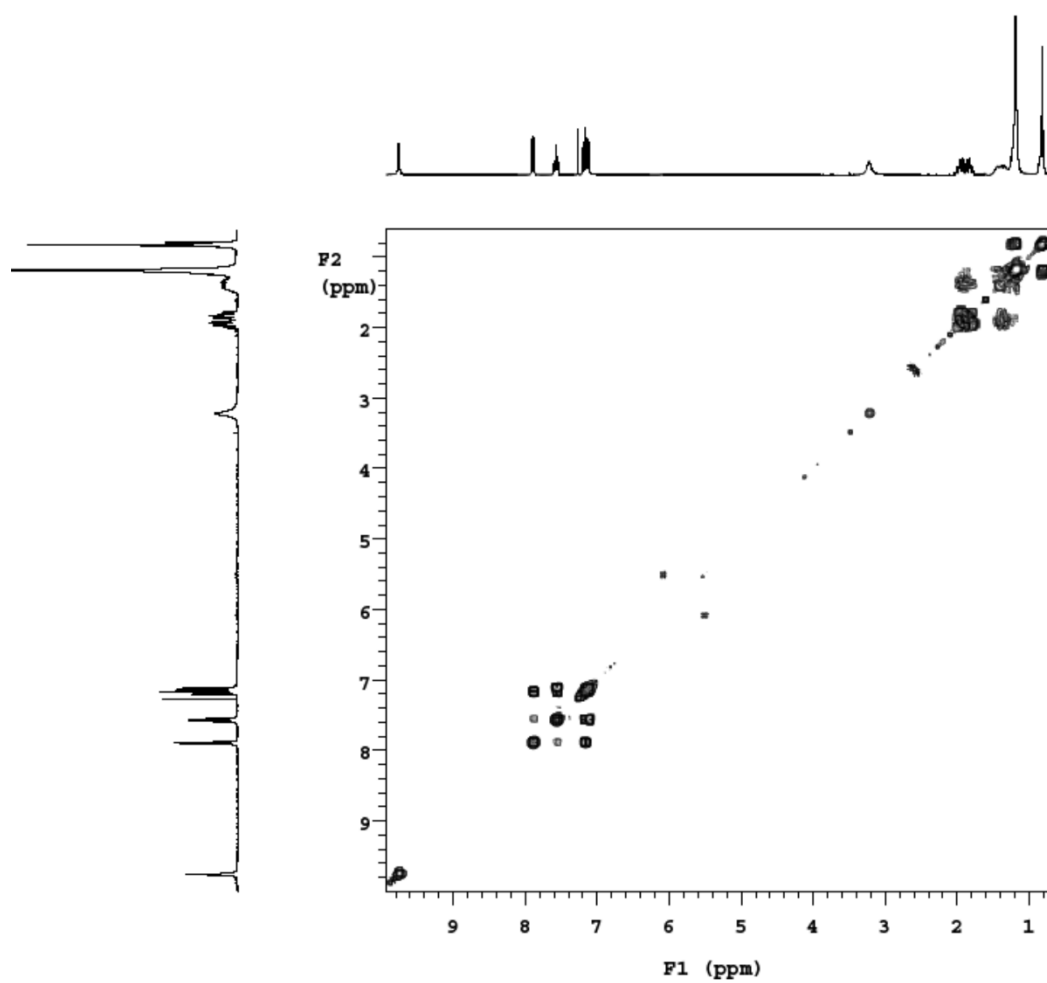

**Figure S3.** COSY spectrum of compound **1** (300 MHz, CD<sub>3</sub>OD)

Supplement: Supplementary file 3 [file Image3.PDF]

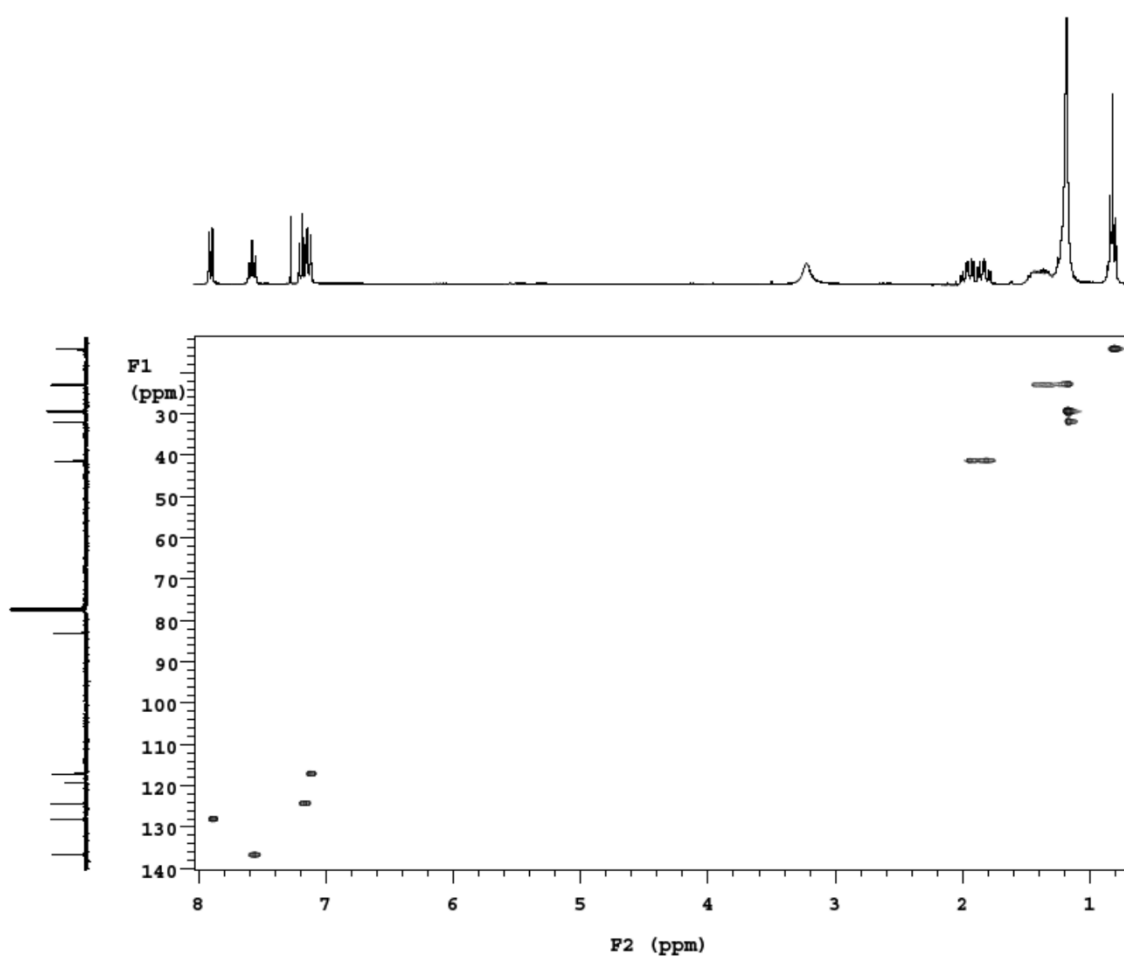

**Figure S4.** HMQC spectrum of compound **1** (300 MHz,  $\text{CD}_3\text{OD}$ )

Supplement: Supplementary file 4 [file Image4.PDF]

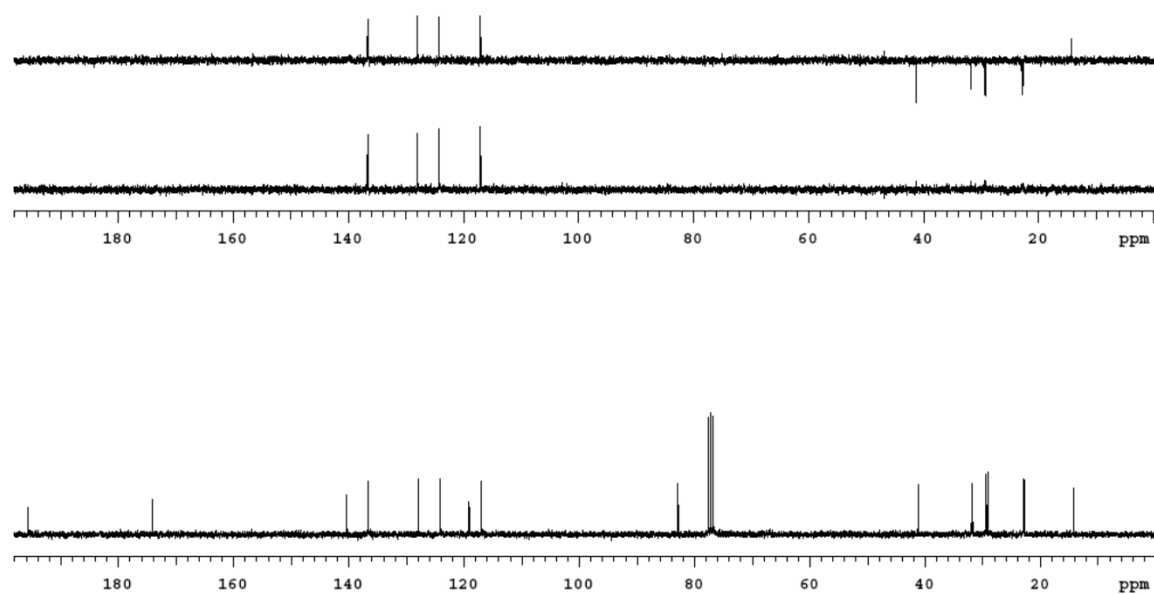

**Figure S5.** DEPT spectrum of the compound **1** (300 MHz,  $\text{CD}_3\text{OD}$ )

Supplement: Supplementary file 5 [file Image5.PDF]

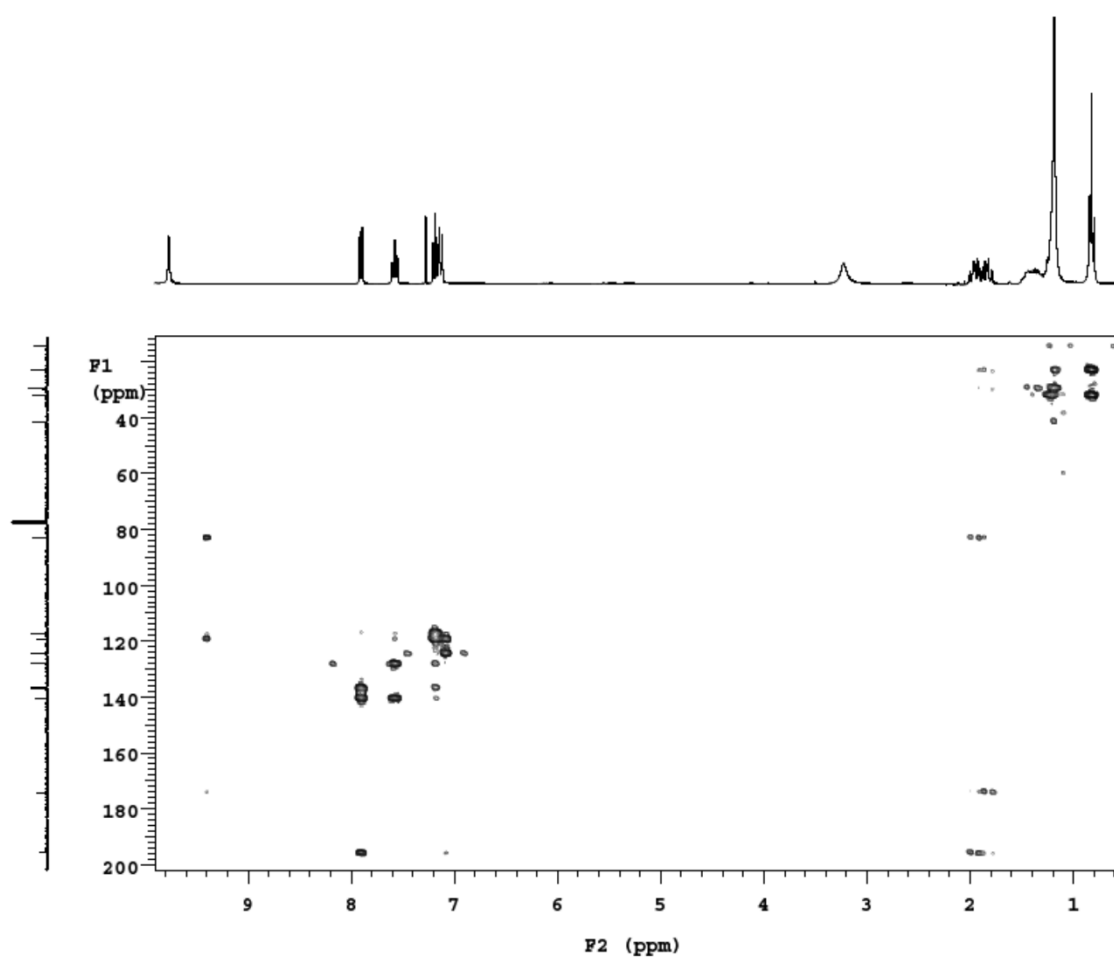

**Figure S6.** HMBC spectrum of compound **1** (300 MHz, CD<sub>3</sub>OD)

Supplement: Supplementary file 6 [file Image6.PDF]

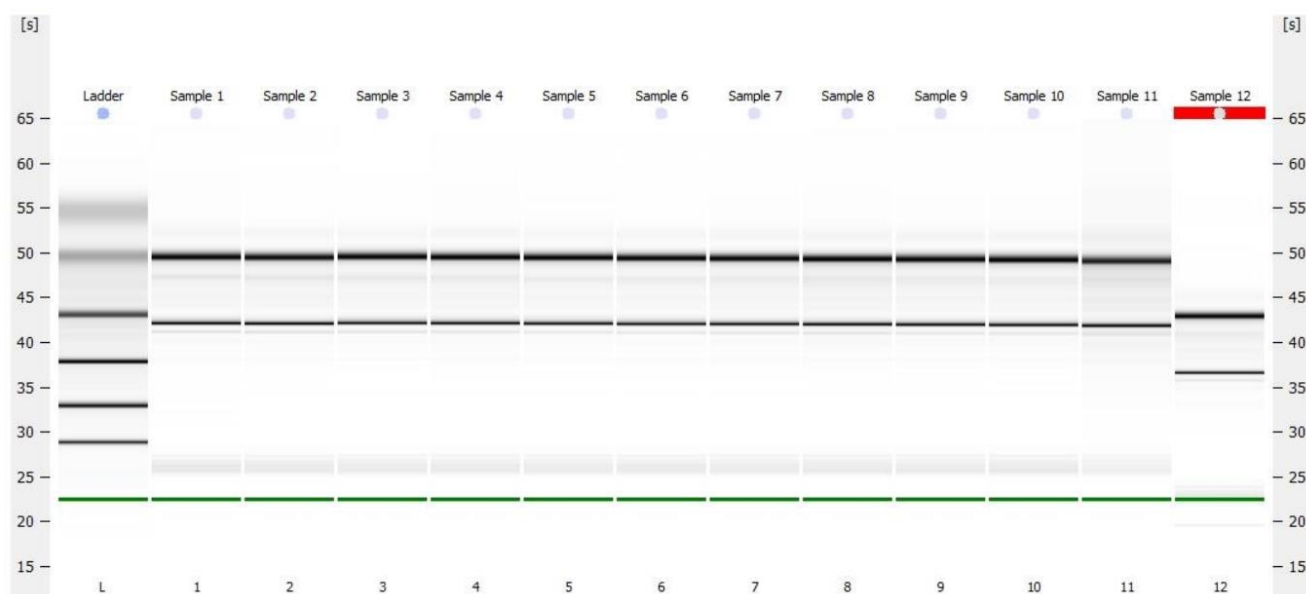

**Figure S7.** Electropherogram of RNA samples obtained after 24 h of cells culture treated.

Supplement: Supplementary file 7 [file Image7.PDF]

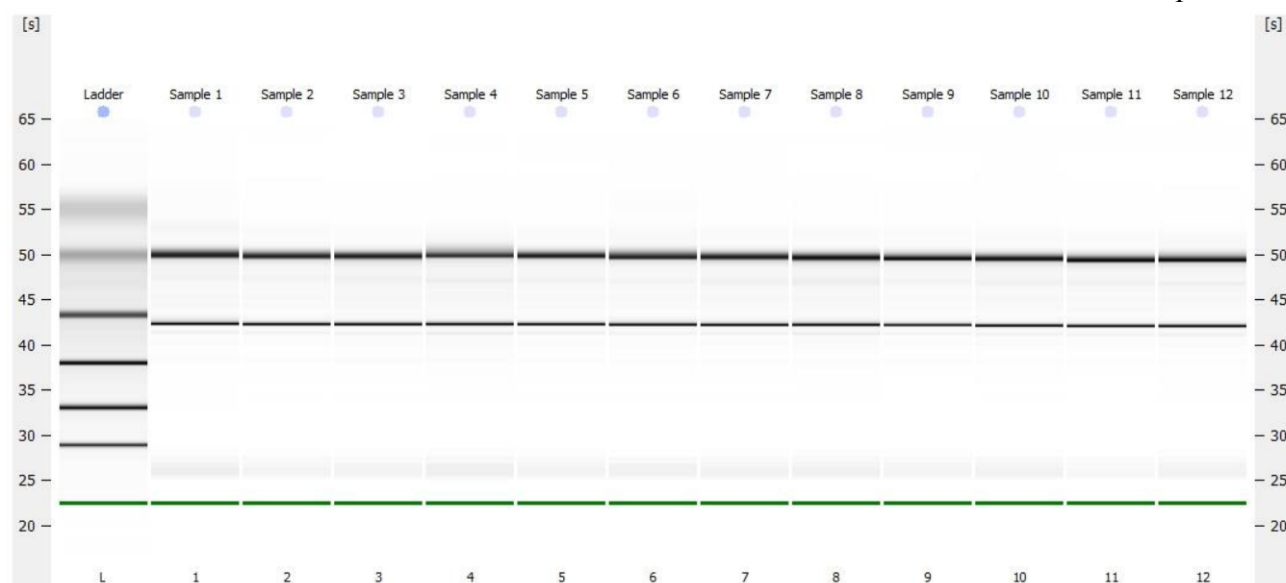

**Figure S8.** Electropherogram of RNA samples obtained after 48 h of cells culture treated.

Supplement: Supplementary file 8 [file Image8.PDF]

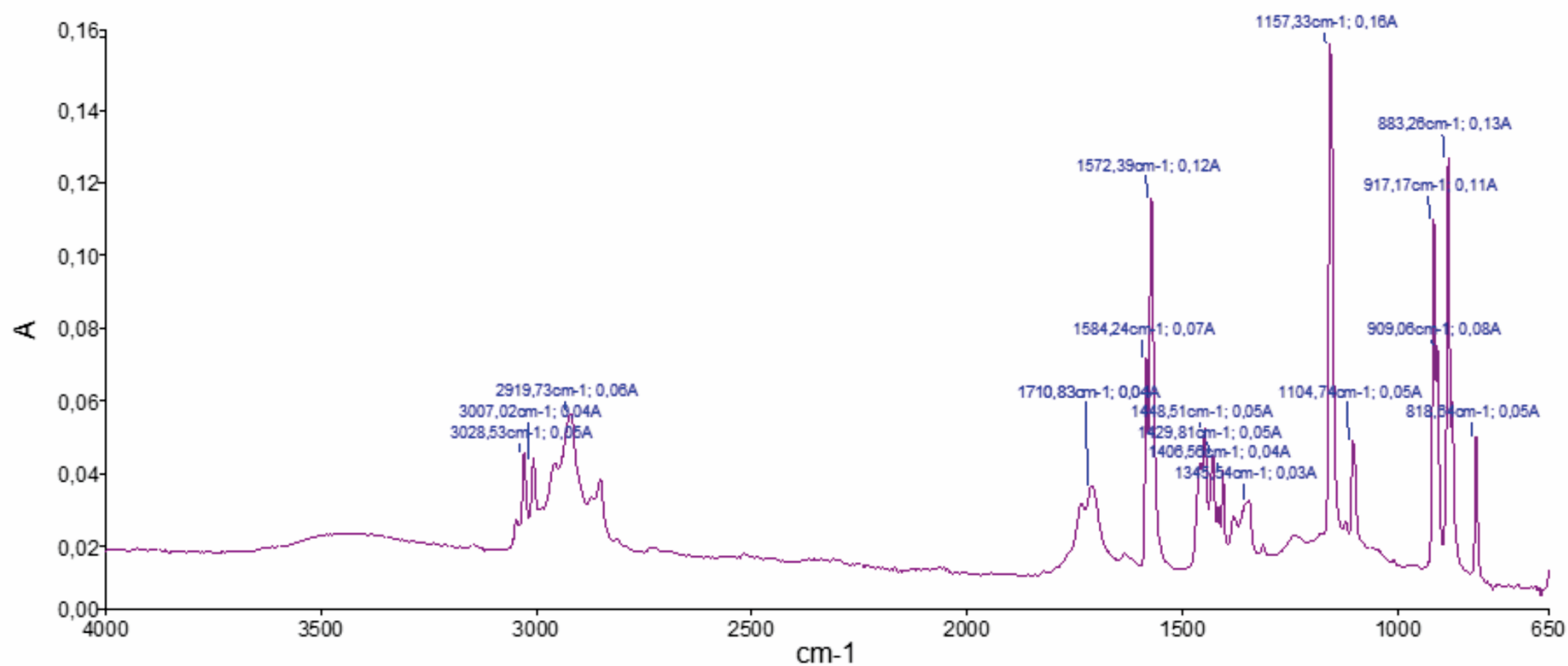

Figure S10. The Infra-Red (IR) of organocopper compound indicating the presence of N at 1,572.32 and 1,584.24 peaks.

Supplement: Supplementary file 10 [file Image10.PDF]
