# Supplementary material for: New Insights about Antibiotic Production by Pseudomonas aeruginosa: A Gene Expression Analysis
Source: Front Chem. 2017 Sep 15;5:66. doi: 10.3389/fchem.2017.00066 (PMC5605626; doi:10.3389/fchem.2017.00066)
Supplement: Supplementary file 9 [file Image9.PDF]

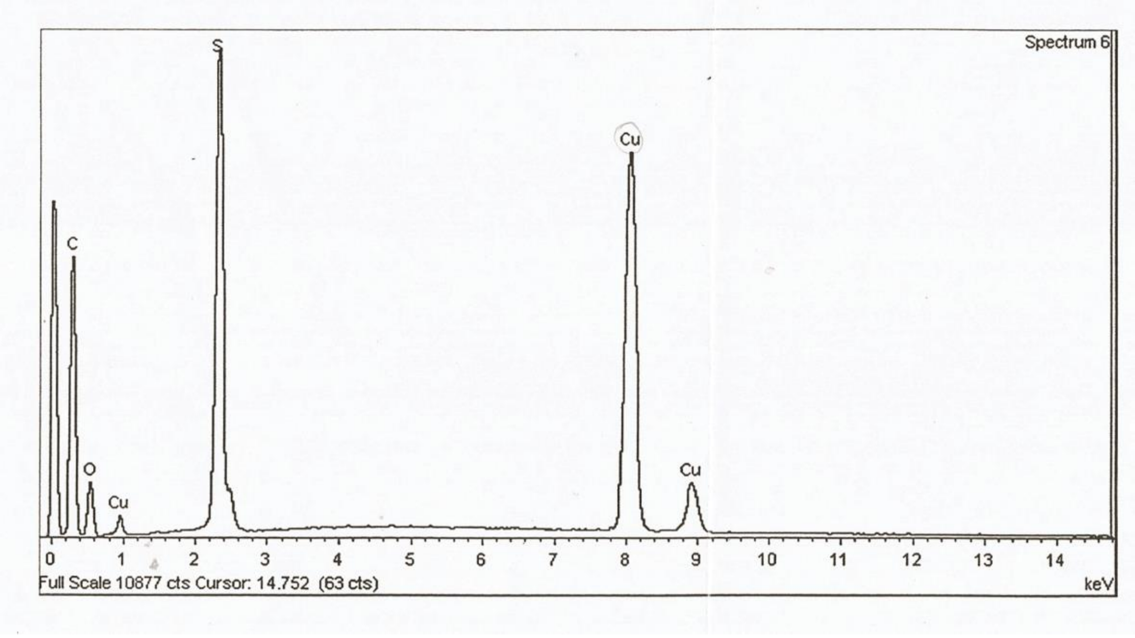

Figure S9: Elemental analysis of organocopper compound by energy-dispersive X-ray espectroscopy (EDS), showing peaks of C, O, Cu and S.
